# The 100 Most-Cited Manuscripts in Hearing Implants: A Bibliometrics Analysis

**DOI:** 10.7759/cureus.33711

**Published:** 2023-01-12

**Authors:** Tsz Ki Ko, Denise Jia Yun Tan, Timothy Shun Man Chu, Jeremy Chan

**Affiliations:** 1 School of Medicine and Surgery, College of Life Sciences, Leicester Medical School, George Davies Centre, Leicester, GBR; 2 Department of Otolaryngology, Nottingham University Hospital, Nottingham, GBR; 3 School of Medicine, Faculty of Medical Sciences, Newcastle University, Newcastle upon Tyne, GBR; 4 Department of Surgery, University Hospitals Coventry and Warwickshire National Health Service Trust, Coventry, GBR

**Keywords:** audiology, cochlear implants, hearing aids, baha, sensorineural hearing loss

## Abstract

The aim of the study was to characterise the most frequently cited articles on the topic of hearing implants. A systematic search was carried out using the Thomson Reuters Web of Science Core Collection database. Eligibility criteria restricted the results to primary studies and reviews published from 1970 to 2022 in English dealing primarily with hearing implants. Data including the authors, year of publication, journal, country of origin, number of citations and average number of citations per year were extracted, as well as the impact factors and five-year impact factor of journals publishing the articles. The top 100 papers were published across 23 journals and were cited 23,139 times. The most-cited and influential article describes the first use of the continuous interleaved sampling (CIS) strategy utilised in all modern cochlear implants. More than half of the studies on the list were produced by authors from the United States, and the Ear and Hearing journal had both the greatest number of articles and the greatest number of total citations. To conclude, this research serves as a guide to the most influential articles on the topic of hearing implants, although bibliometric analyses mainly focus on citations. The most-cited article was an influential description of CIS.

## Introduction and background

Hearing loss affects an estimated 1.57 billion people globally, or more than one in five of the world population, with older people (>50 years) accounting for more than 60% of cases [1]. The causes of hearing loss include hereditary deafness, viral infections and autoimmune diseases [2]. Severe and profound sensorineural hearing loss (SNHL), in which people have minimal functional hearing ability, is present in around 6.7% of the population and can impact severely a person’s psychosocial well-being [3]. The use of technology such as cochlear implants, bone-anchored hearing aids (BAHAs), middle ear implants and auditory brainstem implants (ABIs) to improve hearing can bring both functional and psychosocial benefits [4]. Cochlear implants are the most effective and reliable treatment option for people with SNHL [5]. The development of cochlear implants has been described as one of the most significant technological advances in modern medicine, as they are able to provide people with no functional hearing with the ability to recognise speech and participate more fully in life [6]. The risks of surgery and the potential for side effects need to be carefully considered against the potential benefits of cochlear implantation in deaf adults and children in almost all cases. In children, the use of cochlear implantation can lead to the development of an understanding of grammar and the development of expressive spoken language skills that would be more difficult to acquire later on in life [7]. These implants, developed by Jack Urban in the 1960s and 1970s [8], work by sampling and processing sound and then stimulating the auditory nerve to mimic the output of a normally functioning cochlea. The technology has been gradually improved through the use of more sound channels and processing strategies such as the continuous interleaved sampling (CIS) technique [9-11]. Although cochlear implants are the most widely used hearing implants, other technologies including bone conduction hearing implants (BCHIs), bone-anchored hearing aids (BAHAs) and auditory brainstem implants (ABIs) allow improved hearing in patients with neurological defects, which are not helped by cochlear implants [12].

In the present research, the top 100 most-cited articles from the five decades since their introduction will be presented in a bibliometric analysis. The aim of the study was to characterise the most frequently cited articles on the topic of hearing implants. The objectives of the study are to identify the top 100 most-cited articles on the topic of hearing implants, including the total number of citations and the average number of citations per year since publication, to rank journals that published articles included in the top 100 articles by the number of articles and the total number of citations and to characterise the geographical distribution of studies in the top 100 by country of origin.

## Review

Search strategy

Searches for articles with the greatest number of citations were carried out using the Thomson Reuters Web of Science Core Collection database. The database also includes a Citation Reports tool that provides data for the citation impact, publications per year and citations per year, which could be included in bibliometric analysis. Keywords and synonyms were generated using the study aim, in order for the search to be sensitive to the greatest number of relevant studies on the topic of hearing implants. Boolean search operators (AND and OR) were used to combine search terms to make the search more specific to those studies that would be relevant while excluding irrelevant studies. This strategy was able to maximise search efficiency while reducing the risk of excluding relevant studies. The following Boolean search string was used to search the Web of Science Core Collection database: (((((((ALL=(Hearing implant)) OR ALL=(Cochlear implant)) OR ALL=(Bone anchored hearing aids)) OR ALL=(BAHA)) OR ALL=(Bone conduction hearing implants)) OR ALL=(BCHI)) OR ALL=(middle-ear implants)) OR ALL=(auditory brainstem implants).

Eligibility criteria

Basic eligibility criteria were applied to ensure the studies included were relevant to the aim. The inclusion criteria were that studies were published between 1970 and 2022, were published or available in English, were primary research or reviews and were primarily on the topic of hearing implants. Studies that were published prior to 1970, were unavailable in English and were an editorial or letter or mentioned hearing implants without this being the main focus of the article were excluded from the research. The year 1970 was chosen as a lower limit as the first commercially available cochlear implant was developed in 1972.

Results

A total of 19,047 studies were identified in the initial search. Filters were applied to restrict the search to English language research and primary research and reviews, and within the specified date range. Following the application of filters, 17,090 articles were included. The remaining articles were reviewed manually to ensure that there was a primary focus on hearing implants. Article bibliographic data including authors, year of publication, journal, country of origin, number of citations and average number of citations per year were extracted into a data table. The individual and five-year impact factors for journals publishing the articles were also recorded. Articles were then sorted by the total number of citations and the top 100 articles included in the research.

The most-cited paper included in the research was by Wilson et al. [9], which had 822 citations. This article, entitled ‘Better speech recognition with cochlear implants’, reports the outcomes of the use of cochlear implants with a novel sound processing strategy in seven experienced implant users. Although this study had 133 (+19.3%) more citations than the second-ranked paper by Friesen et al. [13], it had not received the highest average number of citations per year. The fifth-ranked article in terms of the number of citations, also by Wilson et al. [14] and published in 2008, had 52.56 citations per year on average since its publication. The oldest article included in the research was by House [15] and was published in 1978, describing the first successful use of cochlear implants, while the newest article included was a 2014 study by Wanna et al. [16] on the impact of electrode design and surgical approach on cochlear implant outcomes. The years with the greatest number of studies published in the top 100 were 2003 (n=12), 2004 (n=12) and 2005 (n=9). The journal with the greatest number of articles (n=22) and the greatest number of total citations (n=4,789) was Ear and Hearing, a multidisciplinary journal that covers factors affecting the assessment, diagnosis and management of auditory and vestibular disorders.

Although it accounted for very few citations in comparison (n=180), the New England Journal of Medicine had the highest impact factor (91.25) and five-year impact factor (89.68) of any of the journals with articles in the top 100, followed by the Journal of the American Medical Association (JAMA), with 56.27 and 60.15, respectively.

The country with the highest number of articles in the top 100 is the United States of America with 66 articles. The next highest, Australia, had only 13 articles in comparison.

A total of 15 lead authors had more than one article in the top 100 list, with Fu, Gantz and Geers all having three articles each. Of the 100 articles, five were reviews, 13 were proceedings papers and the remaining 82 were primary studies or reports. The top 10 articles cover several different topics including cochlear implant processing strategies, the role of cochlear implants in language development, word recognition following implantation and the role of electrode placement in implant outcomes.

Discussion

The aim of the present research was to characterise the top 100 most-cited articles on the topic of hearing implants through bibliometric analysis. The most-cited paper, by Wilson et al. [9] in 1991, had more than 3.5 times the average number of citations (n = 231) across the whole top 100 articles and 133 (+19.3%) more citations than the second-ranked article. This study describes the development and implementation of the continuous interleaved sampling (CIS) processing strategy for the conversion of ambient sound into electrical stimulation of the auditory nerve. The participants were selected for their good performance with the compressed analogue processor and were assessed using speech reception tests. The authors found that there were large improvements in the speech reception scores of participants following the use of the novel sound processing strategy, which used interleaved sampling to present brief pulses to the implant electrodes in a non-overlapping linear sequence, as opposed to the simultaneous presentation of analogue waveforms to all electrodes in the traditional compressed analogue processor.

The CIS strategy is now used in all commercially available cochlear implants due to its success in allowing open-set word recognition in people with SNHL. As noted by Wilson [6] in a later review, the ability of the brain to make sense of unnatural-sounding inputs and to gradually make greater sense of them over time meant that the job of the implant designer became to present information in a clear enough format, which leads to the development of the influential CIS strategy.

The study with the second highest number of citations (n=689), by Friesen et al. [13] and published in 2001, investigated speech recognition changes when the number of cochlear implant channels was altered. Participants with a Nucleus 22 implant (n=10) and with an Advanced Bionics Clarion implant (n=9) were compared in terms of vowel, consonant, word and sentence recognition. These results were also compared with a control group (n=5) of normal-hearing listeners. The authors used different speech processing strategies (SPEAK, CIS and simultaneous analogue stimulation (SAS)) with an increasing number of spectral channels/electrodes up to a maximum of 20. By increasing the number of channels, the amount of spectral information available to the implant user was increased. The authors found that in the implant users with the best recognition, there were improvements in recognition of sounds as the number of channels increased up to the point of similar performance to normal-hearing subjects. In those participants with the worst hearing at the beginning of the study, there were minimal improvements with more channels. It is likely that this study has been cited so frequently as it demonstrated the potential benefits of adding auditory channels to cochlear implant processing strategies to improve sound recognition.

The study with the third highest number of citations (n=593) was authored by Niparko et al. [17] in 2010. This study was a prospective, longitudinal assessment over three years of spoken language development in a relatively large sample (n=188) of children in the USA who underwent cochlear implantation before five years of age. A control group (n=97) of normal-hearing children was recruited as a comparison. The participants were assessed in terms of their spoken language performance and hearing comprehension at baseline and during the three years of the study. The authors found that there were significantly higher increases in comprehension and expression in children who had undergone cochlear implantation than would be expected from their baseline scores if following a normal hearing development progression. This progress was steeper than for normal-hearing participants, starting from a significantly lower baseline. Implantation at a younger age was associated with significantly steeper improvement, although in the implant group, as a whole, performance in expression and comprehension was still below age-appropriate levels at the end of the three years of follow-up. It is likely that this study has attracted such a high number of citations in the 12 years since its publication due to the significant developmental benefits demonstrated for young children receiving cochlear implants.

The oldest article included in this bibliometric research was by House [15] published in 1976. This was the 81st most-cited article with 171 citations. This is the only article by House in the top 100 most-cited articles, and this has received a high number of citations because it represents a reflection on his development of the first cochlear implant in the late 1960s and early 1970s, which went on to become the basis for future implants. The article is a monograph by House on the origins of his invention in animal studies, followed by a description of the successes and failures of early attempts to create a cochlear implant. The paper also describes the first human participants in his study of cochlear implantation, as well as his predictions about the future of the field and his expectations about the potential of implants to treat profound deafness. As such, this article represents a good ‘first reference’ in reviews of the history of cochlear implantation [8]. The article with the greatest number of citations per year since publication was a review by Wilson and Dorman [14]. This article was a review aiming to provide a history of cochlear implants and present the state of the art in 2008 as well as the limitations of signal processing strategies in use at the time.

The remaining studies in the top 10 and top 100 covered a wide range of topics including speech and language development by children with hearing implants to technical aspects of frequency recognition using cochlear implants. Studies into cochlear implantation, as opposed to other modalities including BAHAs, middle ear implants and auditory brainstem implants, dominate the top 100 citations, which is likely due to cochlear implants being the most widely used device used in SNHL. However, there was a single study into the use of BAHAs by Niparko et al. [17] that investigated their use in the rehabilitation of unilateral deafness. Early studies in the 1970s tended to focus on the technical aspects of basic cochlear implants that affect speech recognition, while those conducted in the 1980s and 1990s tended to investigate improvements in processing strategies. Studies published in the 2000s predominantly focused on the impact of implants on childhood development to a greater extent than in previous decades, while those published since 2010 included studies of new technologies and rehabilitation strategies. The risks of cochlear implants were also addressed in the most-cited articles across decades, including the risk of bacterial infection and complications of surgery. There was also a rising in publications of articles about endoscopic or endoscopic-assisted cochlear implantation in 2022 [18].

The country with the greatest number of citations was the USA. The preponderance of articles from the USA (66%) has been noted in previous bibliometric research, and it is not clear whether this relates to higher levels of output in the USA or the tendency to cite ‘local research’ [19]. It may also reflect the fact that cochlear implants were first developed in the USA and one of the biggest manufacturers of implants is USA-based [20]. The presence of Australia as the country with the second-highest number of citations may also reflect the fact that a major producer, Cochlear Limited, is based in the country [20]. Based on this assumption, it may be surprising that a country with one of the four biggest manufacturers of cochlear implants, France, had so few citations, but this may be due to the limited studies as compared to those published or available in English.

The greatest number of citations came from studies published in the Ear and Hearing journal, which has a relatively low impact factor (1.854 in 2020) and five-year impact factor (3.08). This likely reflects the fact that although this is an important multidisciplinary journal in hearing studies, the field itself is comparatively niche. The journal with the highest impact factor included in the top 100 citations was the New England Journal of Medicine, in which a single study was published by Colletti et al. [21]. Journal impact factor measures the authority and influence of a journal by dividing the total number of citations for articles published in the journal over a two-year or five-year period by the number of citable articles, giving an average number of citations per article. The New England Journal of Medicine is a general medical journal with high standards for submissions aiming to publish the most important and novel medical studies, which are consequently likely to attract more citations than focused publications such as Ear and Hearing, where all studies are on the topic of hearing disorders and therefore has a smaller group of audience. The Journal of the American Medical Association (JAMA), which had four citations in the top 100, is similarly a general medical journal with a high impact factor (2020 impact factor: 56.27, five-year impact factor: 60.15). The journal with the lowest 2020 impact factor (1.49) was Acta Oto-Laryngologica, a journal specialising in otolaryngology and head and neck surgery. As with Ear and Hearing, the target audience is a relatively smaller group of clinicians and researchers, and it is likely that this factor has been criticised as a metric due to the focus on the journal as a whole and for failing to take account of the potentially high impact a single study can have in a specialist journal with a low overall impact factor [22].

Tables and Figures

Table 1 shows the top 100 most-cited articles included in the research ranked by the total number of citations and citations per year [9,13-17,21,23-115].

**Table 1:** Top 100 most-cited hearing implant articles

Rank	First author	Citations	Citations per year
1	Wilson BS [9]	822	26.52
2	Friesen LM [13]	689	32.81
3	Niparko JK [17]	593	49.42
4	Holden LK [23]	473	52.56
5	Wilson BS [14]	417	29.79
6	von Ilberg C [24]	357	15.52
7	Svirsky MA [25]	338	18.78
8	Sharma A [26]	331	19.47
9	Gantz BJ [27]	330	19.41
10	Finley CC [28]	327	23.36
11	van Hoesel RJ [29]	327	17.21
12	Geers AE [30]	326	17.16
13	Kral A [31]	314	31.4
14	Qin MK [32]	292	15.37
15	Blamey P [33]	289	32.11
16	Lee DS [34]	289	13.76
17	Colletti V [21]	285	17.81
18	Turner CW [35]	265	14.72
19	Shepherd RK [36]	264	9.1
20	Kong YY [37]	257	15.12
21	Connor CM [38]	253	15.81
22	Arndt S [39]	251	22.82
23	Fu QJ [40]	250	10.42
24	Stickney GS [41]	248	13.78
25	Stakhovskaya O [42]	247	16.47
26	Ching TY [43]	245	13.61
27	Van de Heyning P [44]	241	17.21
28	Kong YY [45]	241	13.39
29	Nadol JB Jr. [46]	239	9.56
30	Nelson PB [47]	232	12.21
31	Townshend B [48]	232	6.63
32	Aschendorff A [49]	225	15
33	Geers A [50]	223	11.74
34	Zeng FG [51]	223	11.15
35	Dettman SJ [52]	217	14.47
36	Litovsky R [53]	216	13.5
37	Vermeire K [54]	213	16.38
38	Fu QJ [55]	212	12.47
39	Shepherd RK [56]	212	8.48
40	Lazard DS [57]	211	21.1
41	Kiefer J [58]	211	11.72
42	Snik AF [59]	207	12.18
43	Abbas PJ [60]	207	9
44	Rubinstein JT [61]	207	9
45	Gifford RH [62]	206	14.71
46	Nadol JB Jr. [63]	206	9.81
47	Kirk KI [64]	206	7.63
48	Leake PA [65]	204	8.87
49	Richardson RT [66]	202	15.54
50	Vandali AE [67]	202	9.18
51	Nikolopoulos TP [68]	200	8.7
52	Cohen NL [69]	200	6.45
53	Kral A [70]	198	8.25
54	Brown CJ [71]	197	6.16
55	Pisoni DB [72]	196	10.32
56	James C [73]	193	11.35
57	Hinderink JB [74]	193	8.77
58	Gstoettner W [75]	192	10.67
59	Gantz BJ [76]	192	6.62
60	Rosen S [77]	191	8.3
61	Kirk KI [78]	190	8.64
62	Erixon E [79]	187	14.38
63	Henry BA [80]	186	10.94
64	Kiefer J [81]	185	10.88
65	Shannon RV [82]	184	10.22
66	Wanna GB [16]	181	22.63
67	Richardson RT [83]	180	12
68	Reefhuis J [84]	180	9.47
69	Müller J [85]	180	9
70	Firszt JB [86]	179	9.94
71	Brown CJ [87]	179	8.14
72	Ketten DR [88]	179	7.46
73	Skinner MW [89]	178	11.87
74	Fraysse B [90]	178	11.13
75	Nicholas JG [91]	178	11.13
76	Schleich P [92]	178	9.89
77	Dorman MF [93]	176	12.57
78	Xu J [94]	175	7
79	Won JH [95]	174	11.6
80	Rouger J [96]	173	11.53
81	House WF [15]	173	3.76
82	Tobey EA [97]	171	9
83	Fu QJ [98]	171	8.55
84	Morse RP [99]	171	6.58
85	Gantz BJ [100]	170	5
86	Martin BA [101]	169	12.07
87	Cheng AK [102]	169	7.68
88	Peterson NR [103]	167	13.92
89	Litovsky RY [104]	167	10.44
90	Xu L [105]	167	9.82
91	Buchman CA [106]	167	9.28
92	Fayad JN [107]	166	10.38
93	Berlin CI [108]	165	13.75
94	Geers AE [109]	164	8.63
95	Niparko JK [110]	164	8.63
96	Holt RF [111]	161	11.5
97	Cohen LT [112]	159	8.37
98	Shannon RV [113]	158	5.27
99	van Hoesel RJM [114]	157	8.72
100	Wazen JJ [115]	157	8.26

**Figure 1:**
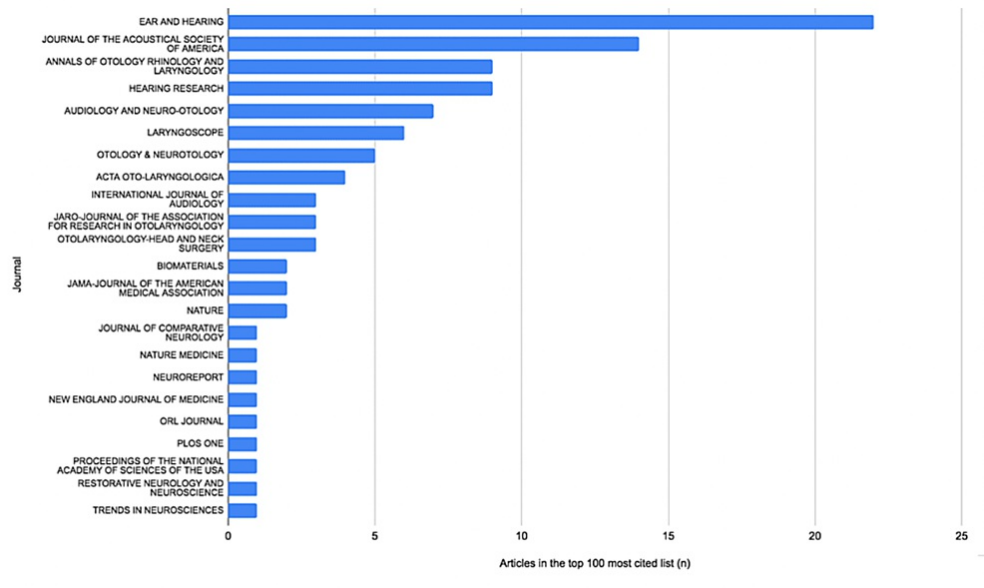
Total articles in the top 100 by journal in which the articles were published

Figure 1 presents the journals in which the top 100 most-cited articles were published, with Ear and Hearing having the greatest number of articles and the greatest number of total citations.

The 2020 and five-year impact factor of journals in which the top 100 most-cited articles were published, as well as the total number of citations for articles, is shown in Table 2, with the New England Journal of Medicine having the highest 2020 and five-year impact factor.

**Table 2:** 2020 and five-year impact factor of journals publishing articles in the top 100, as well as the total number of citations for articles from each journal appearing in the top 100

Journal name	2020 impact factor	Five-year impact factor	Number of citations
New England Journal of Medicine	91.253	89.676	180
Journal of the American Medical Association (JAMA)	56.274	60.151	762
Nature Medicine	53.44	49.248	171
Nature	49.962	54.637	1,111
Trends in Neurosciences	13.837	15.543	314
Biomaterials	12.479	12.104	382
Proceedings of the National Academy of Sciences of the United States of America	11.205	12.291	173
Ear and Hearing	3.57	4.121	4,789
Otolaryngology-Head and Neck Surgery	3.497	3.521	589
Laryngoscope	3.325	3.447	1,214
PLOS One	3.24	3.788	211
Journal of Comparative Neurology	3.215	3.46	204
Hearing Research	3.208	3.719	2,186
Journal of the Association for Research in Otolaryngology (JARO)	3.057	2.996	633
Restorative Neurology and Neuroscience	2.406	3.115	167
Otology & Neurotology	2.311	2.746	1,107
International Journal of Audiology	2.117	2.491	617
Audiology and Neuro-Otology	1.854	3.08	1,564
Journal of the Acoustical Society of America	1.84	2.001	3,691
NeuroReport	1.837	1.726	171
Annals of Otology, Rhinology, and Laryngology	1.547	1.887	1,766
Journal for Oto-Rhino-Laryngology and Its Related Specialties (ORL)	1.538	1.647	357
Acta Oto-Laryngologica	1.494	1.624	780

**Figure 2:**
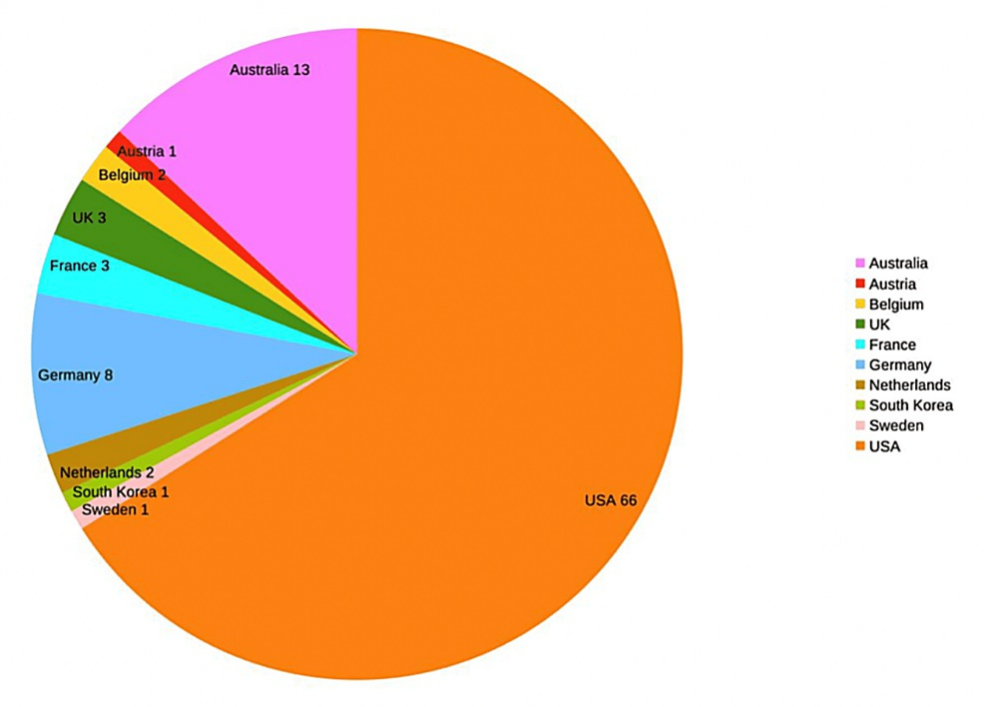
Total citations in the top 100 by country of origin

In Figure 2, the geographical distribution (country of origin) of the top 100 most-cited articles is shown. The USA has the highest number of articles and the greatest number of citations.

Limitations

This research is limited by the flaws in bibliometric analysis as a methodology. The number of citations that an article receives can be influenced by a number of factors that do not necessarily relate to its importance within a field of study. Authors are likely to self-cite their own studies and those of their colleagues. As articles were limited to English language publications, this may also have biased the results towards anglophone countries. Older papers are also likely to receive more citations over time, while important newer articles may have fewer citations simply due to recency bias. These limitations demonstrate that this research should be considered a starting point for investigating important studies into hearing implants but not a definitive statement of the most important 100 articles.

## Conclusions

The most-cited article describes the CIS strategy that has been extremely influential in the development of modern cochlear implants. The majority of the citations came from high-impact peer-reviewed journals, although there were also a number of studies published in small, specialist journals in the top 100. The present research provides a starting point for those wanting to investigate the most influential studies on the topic of hearing implants and what makes a ‘citable’ study.
